# Insular Cortical Thickness in Patients With Somatoform Pain Disorder: Are There Associations With Symptom Severity and Childhood Trauma?

**DOI:** 10.3389/fpsyt.2020.497100

**Published:** 2020-09-10

**Authors:** Elisabeth Meyer, Eva Morawa, Yeliz Nacak, Julie Rösch, Arnd Doerfler, Clemens Forster, Yesim Erim

**Affiliations:** ^1^Department of Psychosomatic Medicine and Psychotherapy, University Hospital of Erlangen, Friedrich-Alexander University Erlangen-Nürnberg (FAU), Erlangen, Germany; ^2^Department of Neuroradiology, University Hospital of Erlangen, Friedrich-Alexander University Erlangen-Nürnberg (FAU), Erlangen, Germany; ^3^Institute of Physiology and Pathophysiology, Friedrich-Alexander University Erlangen-Nürnberg (FAU), Erlangen, Germany

**Keywords:** somatoform pain, insula, cortical thickness, childhood maltreatment, voxel-based morphometry

## Abstract

**Background:**

Studies show significant alterations in insular cortical thickness in patients with somatoform pain disorder (SPD). Additionally, associations between childhood maltreatment and morphometric alterations in insular cortex have been observed. Since patients with SPD often report about adverse childhood experiences, we were interested in the interrelationship of exposure to childhood maltreatment and insular cortical thickness in patients with SPD.

**Methods:**

Fifteen adult patients with SPD (ICD-10 F 45.40/41, DSM-Code 307.80) and thirteen healthy adult controls underwent T1-weighted MR brain imaging. In the voxel-based morphometry (VBM) analysis we compared whole brain cortical thickness between patients and controls using a Student’s two-sampled t-test (*p* < .05). Then we performed a secondary analysis to detect differences in cortical thickness levels in the insular cortex between both groups. For further analysis of differences in insular cortical thickness we used gender, age, depressive symptoms [Patient Health Questionnaire (PHQ)-9], and whole brain cortical thickness as nuisance covariates. Subsequently we explored associations between insular cortical thickness, symptom severity (PHQ-15) and past experiences of childhood maltreatment (CTQ) in both groups.

**Results:**

Patients showed reduced insular cortical thickness in a subregion of right Brodmann area (BA) 13 (anterior part of the insular cortex), whereas whole brain cortical thickness did not differ between groups. The between-group difference in the identified insular subregion of right BA 13 was not diminished by any of the covariates. This implies that the reduction in cortical thickness in the identified insular subregion might be due to a specific group effect. The effect sizes indicate that the group of patients experienced more childhood maltreatment than the control group. Nonetheless, significant correlations of insular cortical thickness with symptom severity and childhood maltreatment in the total collective could not be demonstrated for the group of patients.

**Conclusions:**

Our data suggest that alterations in the identified insular subregion of right BA 13 are associated with somatoform pain, independent of gender, age, or coincident depression levels. To identify significant associations of insular cortical thickness and experiences of childhood maltreatment in patients with SPD investigations within larger samples are highly recommended.

## Introduction

Patients with chronic somatoform pain disorder (SPD) suffer from pain which can not be explained by medical conditions thoroughly (1). Though the underlying mechanisms are not fully understood, a complex interaction of genetic predispositions, epigenetic influences, psychoneuroimmunological and social mechanisms is assumed to be involved in the development of somatoform pain [see (2)].

Previous research reported about differences in cortical thickness of various brain areas between patients with chronic pain disorders and controls (3–7). Although the pathophysiological impact of changes in cortical thickness is not clear yet, studies indicate that a reduction of gray matter is associated with degenerative mechanisms and impairments (8, 9). Several studies focused on changes in cortical thickness especially in the insular cortex in patients suffering from chronic pain (3, 7, 10). The insular cortex shares a wide variety of connections with other pain processing brain areas and in the anterior insula sensory input is liaised with cognitive, motivational, and emotional information from interconnected areas (see (11, 12)). Furthermore, Craig suggests that the anterior insula is involved in interoceptive processing and subjective feelings (13). Given its integrative accomplishment, the anterior insular cortex is said to sub-serve a state of emotional awareness and a condition of selfhood (13–15).

A reduction in gray matter volume - and thus in cortical thickness - might be attributed to overuse atrophy, secondary to excitotoxicity, or influence of inflammatory cytokines (see (16)). For example, in patients with major depressive disorder an elevated interleukin (IL)-6 serum level was associated with reduced prefrontal cortical thickness (17). Frodl *et al*. found that patients with major depressive disorder had a smaller hippocampal volume and showed higher plasma levels of IL-6 and C-reactive protein (CRP) than healthy controls (18). Animal models affirm that neuroinflammation has an effect on neuropathological mechanisms, including neurodegeneration (19) and decrease of neurogenesis (20, 21). Further, studies report about significantly elevated plasma and serum levels of proinflammatory cytokines like IL-6 and IL-8 (22) and elevated IL-8 levels in the cerebrospinal fluid (23, 24) in patients suffering from fibromyalgia. Elevated IL-8 levels in the cerebrospinal fluid were also found in patients with lumbar disc herniation (25), indicating that neuroinflammation plays a role in different forms of chronic pain disorders.

Other evidence suggests an association of early life stress like childhood trauma and pro-inflammatory states in adulthood (26). Early life stress itself comprises a variety of adverse conditions during childhood that are said to have effects on the adult’s mental constitution as well as the behavior, e.g., alcohol abuse or depression (27, 28). Childhood maltreatment is known to be an early life stressor and includes physical and/or emotional ill-treatment, sexual abuse, neglect, and other harmful conditions (29). Patients with somatoform pain disorder and fibromyalgia report about a higher frequency of experiences of maltreatment during childhood than controls with medically explained chronic pain (30). For example, around 32% of patients with somatoform pain disorder or fibromyalgia reported about frequent physical maltreatment during childhood compared to 11% in a control sample and sexual abuse was reported by 10–15% of the patients and 0% by the control group (30). Landa and colleagues suppose a shared physical and social pain neural system that implicates a higher susceptibility to somatoform pain as a result of adverse childhood experiences (2). Other studies consider alterations in cortical thickness as an anatomical correlate of childhood maltreatment (31–33) or a neural marker of increased risk for psychopathology (34). Normally, gray matter volume increases in the period of preadolescence and declines after adolescence, while white matter increases linearly (35). The underlying, well-regulated cellular maturational processes (36) might be disturbed by adverse childhood experiences and result in normative deviation in gray matter volume (see (37)). As delineated above, neuroimmunological processes might be one of the mediating factors between childhood maltreatment and changes in brain morphometry. Changes in gray matter volume which are associated with childhood maltreatment were mainly observed in the gyrus cinguli anterior, frontal and temporal areas, and in the insula (32–34, 38). Furthermore, gyrification deficits in the insular cortex seem to be associated with childhood maltreatment (34).

Since affective disorders and pain syndromes are often coincident, studies investigated the interaction of both on cortical thickness. While some findings point to vanishing differences in cortical thickness in pain syndromes when controlling for affective disorders (4), other studies surmise the concomitance of independent mechanisms (39). The thinning of cortical thickness itself could be associated with deficits in pain regulation and hence promote the manifestation of chronic pain. Alternatively, cortical thinning could be the consequence of continuous nociceptive input. Concerning the cross-sectional design of studies to the topic, both options are conceivable. Furthermore, a competing assumption points to possible increases in gray matter volume due to over-engagement of pain-modulatory functions (6).

Independent branches of research report about alterations in insular cortical thickness in patients with somatoform pain disorder as well as in people with a history of childhood maltreatment. Moreover, it is well established that patients with somatoform pain disorder often made the experience of childhood maltreatment. To our best knowledge, the interrelationship of these variables has not been addressed yet.

We hypothesize that patients diagnosed with somatoform pain disorder express differences in insular cortical thickness compared to healthy controls and that these differences correlate with higher symptom severity. Because studies showed an association between affective disorders and cortical thickness (40), we use depression as nuisance covariate. Furthermore, we postulate that these differences are associated with greater exposure to childhood maltreatment.

## Methods

### Subjects

In the framework of an extensive investigation on somatoform pain disorders (see Nacak et al. (41) for detailed information) 65 patients suffering from somatoform pain disorder were compared with 65 age- and gender-matched healthy controls. Patients and controls were asked to participate in an additional MRI-session in case they were suitable (e.g., free of cardiac pacemaker). Overall 16 patients and 13 healthy controls participated in our MRI-exploration. Retroactively one patient had to be excluded because of damaged MRI-data. At the time of data collection all patients met the criteria for ***“***pain disorder associated with psychological factors***”*** [International Classification of Diseases (ICD)-10 F 45.40/41 (42) or DSM-Code 307.80 (43)]. Thus, the patients sample included persons suffering from chronic pain of any kind (e.g., back pain, headache, fibromyalgia) accompanied by psychological distress and impairments in several functions. In order to verify the patients***’*** diagnoses, the survey made use of the Structured Clinical Interview (SCID-I) for Axis-I-disorders (44). The subsampled control group consisted of 13 healthy persons who were matched for age and gender. Using the short-version of the Structured Clinical Interview for Diagnostic and Statistical Manual of Mental Disorders (DSM)-IV it was reassured that they did not meet the criteria for somatization or mental disorders. All participants were asked to complete the questionnaires described below before neuroimaging.

Exclusion criteria for both participants and control group involved current alcohol or substance abuse, relevant organic disorders or medical preconditions, mental disorders with psychotic symptoms, and language barriers. Additionally, participants younger than 18 years and older than 65 years were excluded from the study.

Informed consent was obtained from all participants and the study was approved by the local ethic committee of the Friedrich-Alexander Universität Erlangen-Nürnberg (approval number: 46_14B).

### Neuroimaging

#### Image Acquisition

High-resolution three-dimensional images were acquired using a 3 T Siemens MAGNETOM Trio Tim scanner (Siemens, Erlangen) with the software version *syngo* MR B17. For the anatomical scans, a T1-weighted sequence with transversal slices and a 12-channel triple mode head coil was used (176 slices per slab; slice thickness = 1 mm; resolution 1 mm^3^; TE = 2.52 ms; TR = 1.900 ms; FA = 9; FoV = 256 mm; FoV phase = 100%; phase resolution = 100%). Images were analyzed with BrainVoyager3.2 (Brain Innovation B.V., Maastricht, the Netherlands; www.brainvoyager.com).

#### Voxel-Based Morphometry Analysis

To make the original data suitable for advanced segmentation, raw data was up-sampled to a 0.5 mm resolution using sinc interpolation. Preparatory steps for cortical thickness measurement were performed using a segmentation tool that separated the cortex from surrounding structures like the cerebellum and subcortical structures and estimated the WM-GM (white and gray matter) and GM-CSF (gray matter and cerebrospinal fluid) boundaries as precisely as possible. These different classes of brain tissues are identified based on the intensity of the signal of each voxel. The according thresholds were adaptively calculated by inspecting local intensity histograms as well as computed gradient fields (for details see (45)).

For the measurement of cortical thickness, the Laplace method as introduced by Jones (46) was used. This method finds the cortical thickness at a certain voxel solving a partial differential equation such that the optimum spatial direction representing the local thickness is found. This is done in a repeated manner from starting at a source voxel (e.g., one at the GM-CSF boundary) and continuing along a transition of intensities. With this, for every GM voxel gradient vectors were computed. The gradient was first followed in one direction and then in the opposite direction. The integrated values of the accumulated step sizes until a boundary voxel was reached resulted in a pathlength. The pathlengths from the two directions were finally added to obtain the thickness measure for the GM voxel.

Each participants’ data was transformed into individual volume maps (IVMs). IVMs represent information of the individuals’ cortical thickness in a voxel-wise level. The whole brain cortical thickness was calculated as the sum of each IVM. With this the whole brain cortical thickness of the patients’ IVMs were compared with that of the control group using a Student’s two-sampled t-test (*p* < .05).

In respect of our *a priori* interest a secondary analysis was performed to highlight differences in cortical thickness levels in the insular cortex between patients and the control group. The respective anatomical localization was determined using the application “MNI/Talairach with Brodmann areas (1.09)” of the Yale BioImage Suite Package (47).

A secondary analysis compared the IVMs between the groups on a voxel based level and highlights brain areas that significantly differ in cortical thickness. A difference in cortical thickness of a certain brain area between the groups was considered if it consists of at least 200 contiguous significant voxels (25 mm³).

### Psychometrics

#### Patient Health Questionnaire (PHQ-15, PHQ-9)

To assess the severity of somatic symptoms the Patient Health Questionnaire (PHQ)-15 module of the German PHQ was used (48). The module includes 15 self-report-items, which conform to the symptoms of DSM-IV somatization disorder. The total PHQ-15 score ranges from 0 to 30 points. Depressive symptoms in patients and controls were evaluated with the PHQ-9 module of the German PHQ (48). A total of nine self-report items coincide the DSM-IV Diagnostic Criterium A symptoms for Major depression disorder. The total PHQ-9 score ranges from 0 to 27 points. Both PHQ-15 and PHQ-9 scores represent mild symptoms from 5 to 9 points, moderate symptoms from 10 to 14 points, and severe symptoms from 15 to 30 (respectively 27) points. German PHQ-15 and PHQ-9 show good psychometric properties (49–51).

#### Childhood Trauma Questionnaire

Experiences of childhood maltreatment were enquired with the 28-item short form of the Childhood Trauma Questionnaire (CTQ) (52) in German language (53). Subscale scores represent the severity of five types of traumatic experiences (physical-, sexual-, and emotional abuse and physical and emotional neglect) and range from 5 (no abuse or neglect) to 25 (maximum of abuse or neglect). Furthermore, a total score from 25 to 125 depicts the gravity of overall maltreatment. The evaluation strategy was based on Wingenfeld and colleagues (54). Three items, which neither contribute to these five subscales nor to the total CTQ score, contribute to the additional subscale minimization/denial. This subscale measures the tendency to trivialize memories (subscale score ranges from 0 to 3 points). The CTQ in German language has good psychometric properties, except for a low internal consistency of the subscale physical neglect (53, 54).

### Statistics

Statistical analyses were conducted with BrainVoyager3.2 and SPSS 25.0 (SPSS Inc., Chicago, Illinois). Descriptive characteristics are represented as mean values, standard deviations, ranges, and frequencies. Sociodemographic and clinical characteristics of the subjects were evaluated with two-sampled t-tests and χ^2^-tests in SPSS (if our data did not fulfill statistical assumptions, we calculated Mann-Whitney-U-tests and Fisher’s exact test instead). PHQ-15 and PHQ-9 were completed by each participant, while CTQ was not filled out by one patient and two healthy controls (i.e., 14/15 and 11/13). Student’s two-sampled t-test provided by BrainVoyager3.2 was used to identify differences in whole brain cortical thickness between groups (*p* < .05). In the subsequent secondary analysis we used Student’s two-sampled t-test to detect subregions in the insular cortex, which showed differences in cortical thickness between patients and healthy controls. These tests are run on a voxel-wise level. For a further statistical analysis we extracted the value of whole brain cortical thickness and the cortical-thickness-values of these identified insular subregions of each participant from BrainVoyager to SPSS. These values were used to calculate the relationship between cortical thickness in the identified insular subregions and somatoform pain disorder while controlling for the level of depression (PHQ-9) and other covariates. For this purpose we performed an analysis of covariance (ANCOVA) with two independent groups and two fixed factors (group and gender). In addition to PHQ-9 scores (i.e., level of depression) we used whole brain cortical thickness and age as additional nuisance covariates, because both parameters are also known to have an influence on cortical thickness. Because ANCOVA is considered to be robust against violations of assumptions rather well, we did not use alternative statistical methods. In a subsequent step we calculated Pearson correlation coefficients of cortical thickness in the identified insular subregions with age, somatic symptom severity (PHQ-15), level of depression (PHQ-9), and experiences of childhood maltreatment (CTQ total and subscale scores). If the assumption of normality was not fulfilled, we calculated Spearman correlation coefficients. The effect size was measured with Cohens’ *d* (55) and *η^2^*. An online tool was used to calculate Cohen’s d and confidence intervals (56). For all analyses the level of significance was predetermined at *p* ≤ .05.

## Results

### Subjects’ Sociodemographic and Clinical Characteristics

For detailed information about the sociodemographic characteristics of our participants, see **Table 1**. Since our sample was age- and gender-matched, patients and healthy controls did not differ by age or distribution of gender. While the control-group was free of chronic pain, patients reported an average pain duration of 10.7 years (SD = 8.8 years). There was no significant difference in pain duration between female and male patients. As delineated above, different types of chronic pain were reported by the group of patients (see **Table 1**). It has to be noted, that some patients even named more than one type of chronic pain (e.g., migraine and fibromyalgia), while other patients did not specify the kind of chronic pain they suffer from. The use of antidepressants and pain medication was only prevalent in the patient group. **Table 2** shows the clinical characteristics of patients and controls. The mean score of somatic symptom severity (PHQ-15) and the degree of depressive symptoms (PHQ-9) was significantly higher in patients compared to healthy controls. The effect sizes referring to the total CTQ-score and the CTQ-subscales indicate that experiences of childhood maltreatment were more occurent in the group of patients than in the control group.

**Table 1:** Demographic characteristics of patients and healthy controls.

Variables	Patients (*n* = 15)	Controls (*n* = 13)	U Score/*χ*^2^	*p*
Age				
Mean ± SD	49.1 ± 11.0	43.0 ± 12.2	61.50	.098^a^
Range	18 − 61	18 − 61		
Gender, n (%)				
Female	11 (73.3%)	9 (69.2%)		>.99^c^
Male	4 (26.7%)	4 (30.8%)	0.06	
Partnership status, n (%)				
Single	5 (33.3%)	7 (53.8%)		.27^b^
In a partnership	10 (66.7%)	6 (46.2%)	1.20	
Educational level, n (%)				
Low (below university entrance diploma)	12 (80.0%)	6 (46.2%)		.11^c^
High (university entrance diploma and/or university degree)	3 (20.0%)	7 (53.8%)		
Employment status, n (%)				
Employed	9 (60.0%)	11 (84.6%)		.22^c^
Unemployed (homemaker/ student/pensioner/jobless/other)	6 (40.0%)	2 (15.4%)		
Pain duration, years				
Mean ± SD	10.7 ± 8.8	–		
Range	2 − 29	–		
Type of chronic pain^d^		–		
Back pain	1	–		
Migraine	1	–		
Fibromyalgia	3	–		
Rheumatoid arthritis	1	–		
Myalgia	1	–		
Not specified	11	–		
Use of antidepressants, n (%)	11 (73.3%)	–		
Use of pain medication, n (%)	9 (60.0%)	–		

^a^Mann-Whitney-U-test, two-tailed; ^b^χ^2^-test, two-tailed; ^c^Fisher’s exact test, two-tailed; ^d^multiple mentions possible.

**Table 2:** Clinical characteristics of patients and healthy controls.

Variable	Patients (*n* = 15)	Controls (*n* = 13)	T Score/U score	*p*	Cohen’s ^*d*^ (95% CI)
PHQ-15, mean ± SD (range)	13.20 ± 4.65 (5–20)	2.08 ± 1.85 (0–7)	**1.50**	**<.001^b^**	**−3.06 (−4.15; −1.97)**
PHQ-9, mean ± SD (range)	13.07 ± 5.31 (5–24)	2.08 ± 1.85 (0–6)	**2.00**	**<.001^b^**	**−2.68 (−3.71; −1.66)**
CTQ total score, mean ± SD (range)^c^	48.57 ± 21.10 (25–89)	35.82 ± 9.05 (26–50)	2.04	.056^a^	−0.75 (−1.57; 0.06)
Sexual abuse, mean ± SD (range)	6.86 ± 4.52 (5–18)	5.55 ± 1.29 (5–9)	73.50	.85^b^	−0.37 (−1.17; 0.42)
Physical abuse, mean ± SD (range)	8.29 ± 5.03 (5–20)	6.18 ± 2.04 (5–10)	61.00	.40^b^	−0.53 (−1.33; 0.28)
Physical neglect, mean ± SD (range)	9.43 ± 4.55 (5–19)	6.64 ± 1.86 (5–10)	48.00	.12^b^	−0.77 (−1.59; 0.05)
Emotional abuse, mean ± SD (range)	10.93 ± 6.37 (5–25)	7.64 ± 2.69 (5–12)	57.50	.29^b^	−0.64 (−1.45; 0.17)
Emotional neglect, mean ± SD (range)	13.07 ± 5.97 (5–23)	9.82 ± 3.60 (5–15)	1.69	.11^a^	−0.64 (−1.45; 0.17)
Minimization, mean ± SD (range)	0.50 ± 0.85 (0–3)	0.18 ± 0.40 (0–1)	62.50	.43^b^	−0.46 (−1.26; 0.34)
Whole brain cortical thickness (ml), mean ± SD (range)	628.15 ± 35.05 (580.29–706.24)	636.12 ± 39.65 (574.06–705.69)	−0.57	.58^a^	0.21 (−0.53; 0.96)

^a^Student’s t test, two-tailed; ^b^Mann-Whitney-U-test, two-tailed; CTQ, Childhood Trauma Questionnaire; GMV, gray matter volume. Significant T scores/U scores and p-values are marked in bold. ^c^The CTQ was only completed by 14 patients and 11 healthy controls. ^d^CI, confidence interval.

### Voxel-Based Morphometry-Analysis

**Figure 1:**
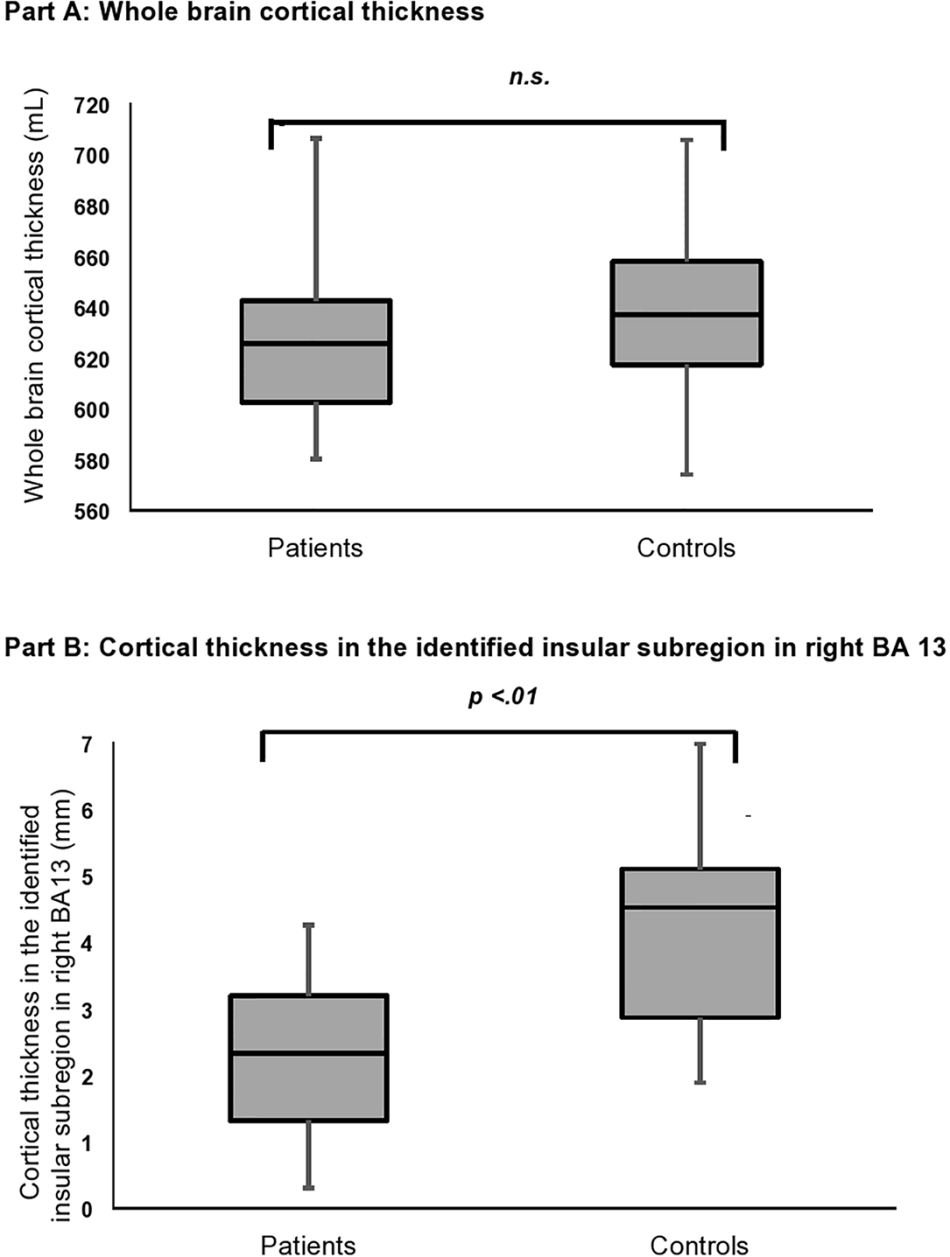
Bar charts represent the whole brain cortical thickness **(A)** and the cortical thickness in the identified insular subregion in BA 13 **(B)** for patients and healthy controls. The median is shown as a line in the center of each box, the bar denotes the first (Q1) and third (Q3) quartile and the whiskers mark the minimum and maximum.

Though patients tend to a smaller whole brain cortical thickness than the control group, the difference was not significant (see **Table 2** and **Figure 1A**). Since it is known that cortical thickness underlies a thinning process, we calculated the correlation between whole brain cortical thickness and age. While there was a slightly significant correlation in the control group between whole brain cortical thickness and age (*r* = −.55, *p* = .051), patients showed no significant association between both parameters (*r* = −.46, *p* = .084). With Student’s two-sampled t-tests in BrainVoyager we could identify one single significant subregion in the insular cortex, which is located in right Brodmann area 13 (X = 36; Y = −8; Z = −4). This subregion is part of the anterior insular cortex and is formed by 225 voxels. As it can be seen in **Figure 1B**, it represents a significant reduction in cortical thickness in the patient group compared to healthy controls [on a voxel-wise level: t (26) = −2.94, *p* = .009]. In this subregion patients showed a mean cortical thickness of 2.27 mm (SD = 1.21 mm; min = 0.31 mm; max = 4.26 mm), whereas healthy controls showed a mean cortical thickness of 4.25 mm (SD = 1.47 mm; min = 1.89 mm; max = 6.98 mm). Just like the whole brain cortical thickness, the cortical thickness in the identified insular subregion is independent of age for both patients (*r* = −.05, *p* = .85) and healthy controls (*r* = −.13, *p* = .66). In a subsequent analysis we compared the mean cortical thickness of the identified insular subregion between groups while using age, whole brain cortical thickness, and depressive symptoms as nuisance covariates. Group and gender were used as fixed factors. In **Table 3**, it is illustrated that the primary between-group effect remained significant.

**Figure 2:**
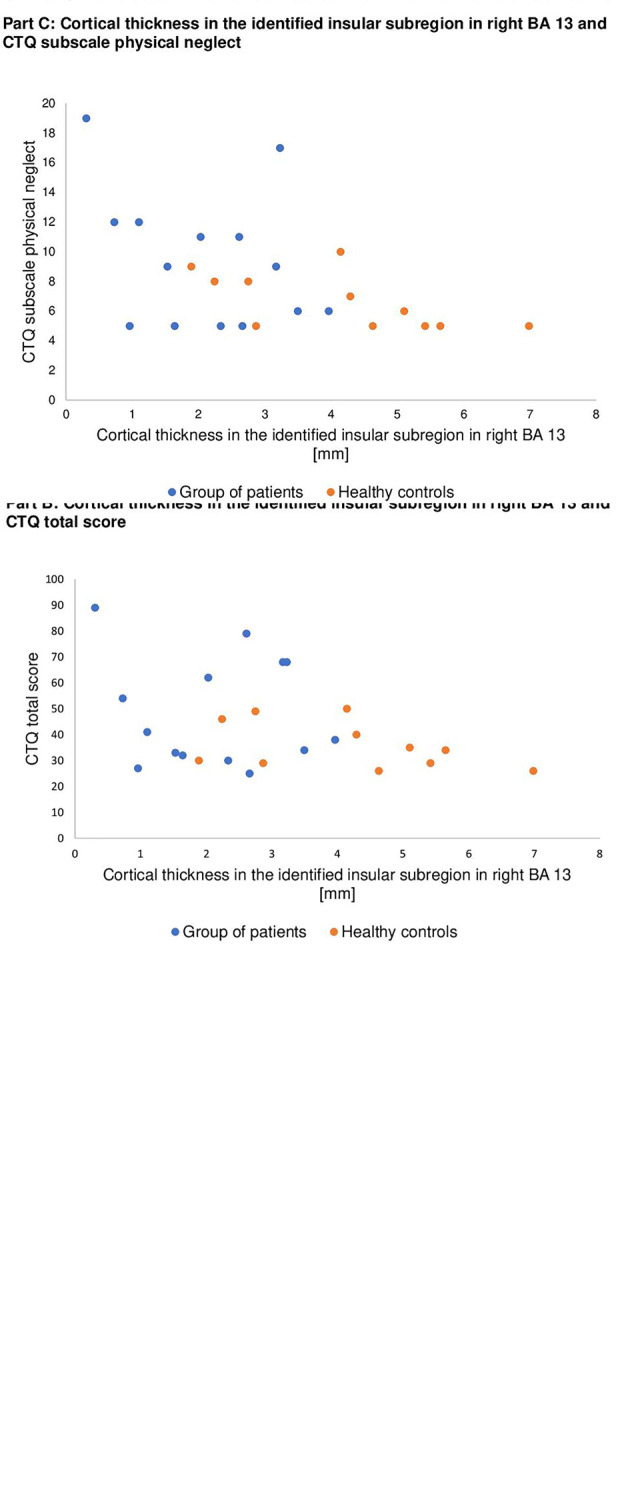
Scatter plots illustrating relationships between cortical thickness in the identified insular subregion in right BA 13 on the x-axis and symptom severity (**Part A**), CTQ total score (**Part B**), and CTQ subscale physical neglect (**Part C**) on the y-axis separately for patients (blue dots) and healthy controls (orange dots).

**Table 3:** Differences in cortical thickness in the identified insular subregion in Brodmann area (BA) 13 between patients with somatoform pain disorder and healthy controls (whole brain cortical thickness, age, and depression as nuisance covariates).

	Mean of squares	*F*	*p*	***η²***
Group effect	9.68	4.97	**.037**	**.19**
Gender	2.95	1.51	.23	.07
Group effect x gender	0.69	0.35	.56	.02
Whole brain cortical thickness	0.61	0.32	.58	.02
Age	0.84	0.43	.52	.02
Depressive symptoms (PHQ-9)	0.86	0.44	.52	.02

PHQ, Patient Health Questionnaire; F, F score of the analysis of covariance (ANCOVA). Significant p-values are marked in bold.

### Association of Insular Cortical Thickness With Somatic Symptoms and Traumatic Childhood Experiences

Correlation coefficients between cortical thickness in the identified insular subregion with age, level of depression (PHQ-9), somatic symptoms (PHQ-15), and traumatic childhood experiences (CTQ) are presented in **Table 4** for the entire sample and in the right column for the group of patients. Negative coefficients represent more somatic symptoms and more traumatic childhood experiences in persons with less cortical thickness in the identified insular subregion. The relationship between cortical thickness in the identified insular subregion in right Brodmann area (BA) 13 and symptom severity (PHQ-15), CTQ total score and CTQ subscale physical neglect is visualized in **Figures 2A–C**.

**Table 4:** Correlation-coefficients of cortical thickness in the identified insular subregion in Brodmann area (BA) 13 (X = 36; Y = −8; Z = −4) with somatic symptoms severity and traumatic childhood experiences.

	Whole sample *n = 28*		Patients *n = 15*	
	*r*	*p*	*r*	*p*
Age	−.31^b^	.11	−.05^b^	.85
PHQ-15	−**.65^b^**	**<.001**	−.39^a^	.15
PHQ-9	−**.50^b^**	**.007**	.27^a^	.32
CTQ total score	−.26^b^	.21	−.10^a^	.73
CTQ physical abuse	−.38^b^	.064	−.32^b^	.27
CTQ emotional abuse	−.15^b^	.47	.06^b^	.83
CTQ sexual abuse	.07^b^	.73	−.00^b^	.99
CTQ physical neglect	−**.45^b^**	**.023**	−.27^b^	.35
CTQ emotional neglect	−.20^a^	.34	.10^a^	.72
CTQ minimization/denial	−.25^b^	.24	−.52^b^	.060

^a^Pearson’s r, two-tailed; ^b^Spearman ρ, two-tailed; PHQ, Patient Health Questionnaire; CTQ, Childhood Trauma Questionnaire. Significant correlation-coefficients and p-values are marked in bold.

## Discussion

The aim of the study was to investigate whether insular cortical thickness differs between patients with somatoform pain disorder and healthy controls. Using voxel-based morphometry we identified a local reduction in cortical thickness in the anterior insular cortex in patients, though the whole brain cortical thickness was the same in both groups. Furthermore, we were interested in associations between insular cortical thickness, symptom severity, and experiences of childhood maltreatment. Whereas significant correlations between insular cortical thickness, symptom severity, and childhood maltreatment were observed in the total collective, associations in the group of patients missed significance.

### Insular Cortex

Previous research on insular cortical thickness alterations in pain disorders have shown ambiguous results. Most studies report about a decline in insular cortical thickness and other pain areas in chronic pain syndromes (3, 7, 10). In contrast, Ceko showed an age-dependent increase in insular cortical thickness in younger patients with fibromyalgia (6). The authors surmised the increment in cortical thickness to reflect an adaptive coping mechanism, which exhausts with increasing age. They suggest that this process results in a reduction of insular gray matter volume only present in older patients, but not in younger patients or healthy controls. Furthermore, the influence of affective disorders on cortical thickness and possible interactions with chronic pain have been discussed controversely (4, 7, 39). With this investigation, we intended to evaluate the mere relation of somatoform pain and insular cortical thickness, independent of age, and depressive symptoms. For this purpose, we tried to eliminate the influence of age and depression by treating them as nuisance covariates. Our data indicate that insular cortical thickness is reduced in patients compared to healthy controls, independent of effects of age or depression.

#### Age

We found that whole brain cortical thickness, which does not differ between groups, is not associated with age. The same accounts for the identified insular subregion in BA 13. The observation, that insular gray matter volume is age-dependent in patients with fibromyalgia (6), could not be confirmed in our sample, neither for patients nor for healthy controls. Though missing significance, the correlation of whole brain cortical thickness with age was considerably higher compared to the correlation of cortical thickness in the identified insular subregion. This could render a hint that insular cortical thickness might be more dependent on other sources of influence than on aging mechanisms, whereas other brain regions are more prone to senescence.

#### Depressive Symptoms

Patients reported significantly more depressive symptoms than healthy controls. Because the influence of affective disorders on brain morphometry is well known, we entered depression in our analysis as a covariate. When Hsu and colleagues controlled for affective disorders, differences in insular gray matter volume between patients with fibromyalgia and healthy controls diminished (4). We tried to question this by using depressive symptoms as additional nuisance covariate and found that the group effect remained significant. This finding supports other surveys which assume that both pain and affective disorders exert influence on insular morphometry, but each with individual and differing mechanisms. So far, the significant correlation of cortical thickness in the identified insular subregion with depressive symptoms in the total collective affirms the recommendation to consider level of depression in investigations about cortical thickness.

#### Symptom Severity

Patients showed a significantly higher symptom severity than controls. This observation is not surprising given that the control group does not conform to the clinical diagnosis of chronic pain disorder. Nonetheless, occasional experiences of pain were reported by healthy controls as well (see PHQ-15 score in **Table 2**). We observed a strong negative association between symptom severity and cortical thickness in the identified insular subregion in the total collective (i.e., cortical thinning in this subregion correlates with higher symptom severity). Referring to the outstanding role of the anterior insular cortex in pain processing, we assume that the ability to deal with pain deteriorates with decreasing cortical thickness in the anterior insular cortex. If the gray matter volume in the anterior insular cortex falls below a critical value, the anterior insular cortex might lose essential abilities in pain processing mechanisms which results in a condition of chronic pain. There is also a negative association between symptom severity and cortical thickness in the identified insular subregion in the patient group, but the correlation missed significance. Maybe this relation should be verified within a larger sample. Alternatively, the reason why we failed to proof an association could be due to the gender characteristics of our sample. In contrast to our gender-mixed sample, most data about fibromyalgia and somatoform pain were collected within all-female samples. This is relevant to the topic, because females are said to report more physical symptoms than men (57, 58). One possible explanation might be that a higher “attentiveness to altered body states” inheres in women (59). Concerning that, the rate of symptom severity and consequently the correlation of cortical thickness and symptom severity might be underestimated in our survey compared to studies with exclusively female patients.

### Childhood Maltreatment and Insular Cortical Thickness

A central question of this investigation refers to associations between experiences of childhood maltreatment and changes in insular cortical thickness in patients with somatoform pain disorder. As delineated above, patients with pain syndromes often faced traumatic experiences during childhood or adulthood (30, 60, 61). Presumably mediated by intensified neuroinflammatory processes, changes in brain morphometry might be promoted. In turn, alterations in brain morphometry like reduced cortical thickness in specific brain processing areas might increase the vulnerability to the development of somatoform pain disorders. It has to be noted that previous research focused mainly on prefrontal and temporal regions, as well as the gyrus cinguli anterior. Until now the association between insular cortex morphometry and adverse childhood experiences has been examined in very few investigations (33, 34). Referring to the medium-to-large effect sizes our data support the assumption that patients with somatoform pain disorder were more often exposed to childhood maltreatment than the comparative group of healthy persons. Only for two subscales (sexual abuse and minimization) the effects were low. To increase the statistical power and the validity of results it is highly recommended that further research should be undertaken in larger samples. Besides to that we assume the way we assessed childhood maltreatment might be not sufficient to the question. Commonly, one main problem in asking adults about experiences of childhood maltreatment is the assessment in retrospect. Especially false negative reports are frequent recall problems (62) and the current psychic and mental status can influence memory processing and fuel recall biases (see (63)). To heighten the accuracy of retrospective reports about childhood experiences, future research could use biographical interviews, interviews of siblings, or similar in addition to questionnaires.

Furthermore, we were interested in possible associations between cortical thickness in the identified insular subregion and exposure to childhood maltreatment. The results of our investigation could not strengthen the assumption about a negative association of cortical thickness in the identified insular subregion and the experience of childhood maltreatment, except for the subscale physical neglect: In the total collective we found a significant negative correlation between physical neglect and cortical thickness in the identified insular subregion in BA 13. This result implies that people with a greater experience of physical neglect during childhood might show a decline in cortical thickness in the anterior part of the insula. As the anterior insular cortex is said to provide services of interoception and a state of self-awareness (see (13)), we suggest that a deficit of physical stimulation might reduce the informational input for the anterior insular cortex in a sensitive developmental period. That might result in reduced local gray matter volume and maybe subsequently compromise the ability to deal with experiences of pain. This assumed interrelation might play a critical role in the multifactorial developmental mechanisms of chronic pain disorders. Nonetheless it has to be considered, that the internal consistency of the subscale physical neglect is rather weak (53) and that we could not confirm associations of physical neglect and cortical thickness in the identified insular subregion for the group of patients. Since only 14 patients completed the questionnaire about childhood maltreatment, it would be expedient to repeat our investigation within a larger sample.

### Strengths and Limitations

Our study targets on linking insular cortex morphometry with childhood maltreatment in patients suffering from somatoform pain disorder. To our best knowledge, this is the first time that this specific interrelation is explored. One strength of our study is the accurate matching of patients and healthy controls for age and gender. Although we did not match our samples for further characteristics, there were no significant differences in educational level, partnership or employment status between groups (see **Table 1**). Furthermore, we tried to minimize the impact of factors which are known to affect cortical thickness themselves. That’s why we included gender, whole brain cortical thickness, age, and depressive symptoms in our exploration. Hence, the reduction in cortical thickness in the identified insular subregion in BA 13 in patients with somatoform pain disorder most probably represents a specific group effect.

Notwithstanding the generalization of our results is limited by some factors. First, the main weakness of our study is the relatively small sample size and therefore low statistical power. Second, most data about fibromyalgia and somatoform pain was collected within mere female samples. As women report more physical and somatoform symptoms compared to men (57, 58), our results might be rarely comparable with other investigations. Nonetheless we assume investigations with mixed samples to be reasonable, because somatoform pain disorders affect both women and men. Another limiting factor is that we did not consider a systematic influence of medication usage on our analyses. Although we asked the participants to pause pain medication and intake of antidepressants for 24 h prior to the MRI-session, we cannot estimate the influence of prolonged effects of the medication on brain imaging. Since pain medication intake and usage of antidepressants are assumed to affect gray matter volume (64, 65), it might be possible that our results are associated with medication-related effects. Another limitation of our exploration is the way how childhood maltreatment was assessed. As explained above, retrospective evaluations are often superimposed with falsification. Hence, it is difficult to rate the accuracy of the reports. Finally, the major limitation of our study is up to its cross-sectional design. We need prospective research to review the causal direction and complex relation of changes in cortical thickness, childhood maltreatment and somatoform pain disorder.

### Implications for Future Research

Taking into account current findings about the role of proinflammatory cytokines in developmental and maintaining processes of chronic pain, it would be interesting to put relevant markers in serum and/or cerebrospinal fluid in relation to neuroanatomical characteristics and experiences of childhood maltreatment in the target group. Considering and examining as many relevant factors in the etiology of somatoform pain disorder as possible in future research will help to establish appropriate diagnostic and therapeutic tools for patients affected.

## Author’s Note

The present work was performed in fulfillment of the requirements for obtaining the degree “Dr. med.”" for author Elisabeth Meyer.

## Data Availability Statement

The raw data supporting the conclusions of this article will be made available by the authors, without undue reservation, to any qualified researcher.

## Ethics Statement

The studies involving human participants were reviewed and approved by Local ethic committee of the Friedrich-Alexander Universität Erlangen-Nürnberg (approval number: 46_14B). The patients/participants provided their written informed consent to participate in this study.

## Author Contributions

YE and YN designed the study. AD and JR provided the MRI data. CF instructed ElM during the preparation and evaluation process of the MR images. EvM supported ElM with the statistical analyses. ElM wrote the first draft of the manuscript. YE, CF and EvM helped in finalizing the manuscript.

## Conflict of Interest

The authors declare that the research was conducted in the absence of any commercial or financial relationships that could be construed as a potential conflict of interest.

## Abbreviations

**SPD**, somatoform pain disorder; **ICD**, International Classification of Diseases; **DSM**, Diagnostic and Statistical Classification of Mental Disorders; **MRI**, magnetic resonance imaging; **VBM**, voxel-based morphometry; **PHQ**, Patient Health Questionnaire; **CTQ**, Childhood Trauma Questionnaire; **BA**, Brodmann area; **IL**, interleukin; **CRP**, C-reactive protein; **3T**, 3 Tesla; **GM**, gray matter; **WM**, white matter; **CSF**, cerebrospinal fluid; **IVM**, individual volume maps.
